# Cost-Effectiveness Analysis of Psoriasis Treatment Modalities in Malaysia

**DOI:** 10.15171/ijhpm.2019.17

**Published:** 2019-04-06

**Authors:** Nor Azmaniza Azizam, Aniza Ismail, Saperi Sulong, Norazirah Md Nor

**Affiliations:** ^1^ Department of Community Health, Faculty of Medicine, Universiti Kebangsaan Malaysia, Selangor, Malaysia.; ^2^ Faculty of Business and Management, Universiti Teknologi MARA Selangor, Selangor, Malaysia.; ^3^ Department of Community Health, Universiti Kebangsaan Malaysia Medical Centre, Kuala Lumpur, Malaysia.; ^4^ Medical Department, Universiti Kebangsaan Malaysia Medical Centre, Kuala Lumpur, Malaysia.

**Keywords:** Cost-Effectiveness, Psoriasis, Phototherapy, Systemic, Biologic, Malaysia

## Abstract

**Background:** There is limited evidence detailing the cost-effectiveness of psoriasis treatments in the Asian region. Therefore, this study is aimed to evaluate the cost-effectiveness of 3 psoriasis treatments tailored for moderate to severe psoriasis, namely topical and phototherapy (TP), topical and systemic (TS), and topical and biologic (TB) regimens, respectively.

**Methods:** This has been achieved by the participation of a prospective cohort involving a total of 90 moderate to severe psoriasis patients, which has been conducted at 5 public hospitals in Malaysia. The main outcome measures have been evaluated via cost and effectiveness psoriasis area severity index (PASI)-75 and/or body surface area (BSA) <5 and/or dermatology life quality index (DLQI) ≤5), estimated from the societal perspective over a 6-months duration. All costs are based on 2015’s recorded Malaysian Ringgit (RM) currency.

**Results:** Consequently, TS has been found to be the most cost-effective treatment with the lowest cost/PASI-75/and/or BSA <5 and/or DLQI ≤5, valued at RM9034.56 (US$2582.55). This is followed by TP, which is valued at RM28 080.71 (US$8026.93) and TB, valued at RM54 287.02 (US$15 518.06). Furthermore, one-way sensitivity analysis has highlighted the cost of medication as the most sensitive parameter.

**Conclusion:** Thus, the input from this study is helpful for policy-makers in determining the first line treatment for moderate to severe psoriasis with consideration of the costs and its effectiveness in Malaysia. This will consequently allow hospitals to justify and provide the essential resources for further research and development, as well as the adoption of better treatment options

## Background

Psoriasis is a skin disease characterized by a dry and thick silvery scaling on its surface. Occurring worldwide, it affects 7.5 million Americans equivalent to 2% to 4% of its population,^1^ 2.8% of the UK population,^2^ 0.19% to 0.24% in Taiwan, and 0.4% in China.^3^ In Malaysia specifically, a total of 17 071 patients with psoriasis from 24 dermatology centres (20 government hospitals, 2 private centres and 2 university hospitals) were registered in Malaysia Psoriasis Registry during the period of 2007 until 2016.^4^ For a majority of the cases, it typically begins at the age of 20-35 years old and synonymous with a paramount effect upon the quality of one’s life, comparable to other chronic diseases like cancer, hypertension, heart disease and diabetes.^5,6^ Psoriasis is also associated with various comorbidities, such as non-alcoholic fatty liver disease (ie, the most prevalent comorbidity in western countries),^7^ obesity, hypertension, dyslipidaemia and diabetes,^8,9^ and mental illness.^10^

Therefore, various treatment regimens have been outlined for cases of moderate to severe psoriasis, which includes phototherapy, systemic and biologic methods. In many cases, topical agents are generally used as co-medications to reduce the side effects and enhance the effectiveness of the treatments. Then, phototherapy utilises UV light to absorb into the skin and reduce cell proliferation, and induce T cells and keratinocyte apoptosis.^11,12^ Meanwhile, systemic medications are prescription drugs that affect the entire body and given to patients who are nonresponsive to phototherapy.^6^ These systemic agents and its usage should be decided with consideration of their dosage, safety and side effects.^13,14^ In contrast to systemic treatment, biologic treatment functions by reducing symptoms of the disease by targeting a specific immune pathway. Commonly considered as the best discovery in the management of moderate to severe psoriasis, most biologic agents have demonstrated high safety profiles without causing toxicity in the organs.^15,16^ These agents are widely used in Spain, with 19.4% of the total psoriasis patients being prescribed with it, followed by the United Kingdom (9.1%), and France (8.4%).^17^ However, the number of patients receiving this type of treatment is still limited in Malaysia.

The different treatment modalities are distinguishable due to their significant effects on the overall cost. Despite being highly efficacious, biologic therapy is particularly attributable to sizable incremental costs, thus resulting in a considerable financial impact.^18,19^ For systemic treatment, the overall cost of the treatment is increased by the need for screening and monitoring tests to be done prior so as to identify any risks of toxicity developing. Meanwhile, phototherapy is particularly limiting as it causes significant loss of productivity due to patients who may have to take off days to get their treatment at the outpatient clinic, amounting to twice or 3 times a week. An estimated 15%-20% of patients have reported to experience reduced of working ability,^20-23^ whereas a staggering 49% of them have missed their working days due to psoriasis.^24^

Given the considerable economic impact of psoriasis towards patients and hospitals alike, this has rendered an economic analysis comparing the cost-effectiveness of these treatment modalities to be imperative. However, most of the available studies are reported to be either of low quality and short time duration, using non-comparable effectiveness measures, recorded incomplete cost calculation, or lacks a sensitivity analysis.^25^ Moreover, differences in methodological criteria for the studies have also yielded inconclusive findings. To date, no study has yet attempted to measure the cost-effectiveness of psoriasis treatment modalities in this region. Thus, the objective of this study is to evaluate the cost-effectiveness of 3 psoriasis treatment modalities from the societal perspective, namely: topical and phototherapy (TP), topical and systemic (TS), topical and biologic (TB).

## Methods

### Design and Setting

A prospective cohort study has been conducted in 5 public hospitals (ie, Universiti Kebangsaan Malaysia Medical Centre, Hospital Kuala Lumpur, Hospital Pulau Pinang, Hospital Sultanah Bahiyah, and Hospital Sultanah Aminah) involving a total of 90 psoriasis patients between January 2016 until March 2017.

The inclusion criteria were moderate to severe psoriasis (psoriasis area severity index [PASI] >10 and/or body surface area [BSA] >10 and/or dermatology life quality index [DLQI] >10), which was similar to the local and international guideline for the management of psoriasis vulgaris,^4,26,27,55^ sought treatment (TP, TS, and TB) at the study settings between January 2016 until August 2016, aged 18 years and above and Malaysian citizenship. The choice of treatment was made based on clinical criteria, without randomization. Mild psoriasis patients were excluded.

### Measure of Effectiveness

Effectiveness was measured based on the PASI, BSA, and DLQI scores. The specific indicator of effectiveness was PASI-75 (75% improvement over the baseline score) and/or BSA <5 (affected area has reduced) and/or DLQI ≤5 (disease has minimal impact on quality of life), 6 months after treatment is initiated.

### Cost Analysis

An economic evaluation was conducted to calculate costs associated with the management of moderate to severe psoriasis, effectiveness and cost-effectiveness of all 3 modalities over a period of 6-months. Discounting for future costs and results was not applied in cost analysis because both cost and effective outcomes occurred at the same period of time (maximum of 6 months).^12^ The total costs of managing moderate to severe psoriasis were calculated from societal’s perspective. All costs were presented in Malaysian Ringgit (RM) 2015. From provider’s perspective, cost of medication, lab tests and radiology were included. Meanwhile, patient’s costs include out-of-pocket expenses and transportation (direct cost) and loss of productivity (indirect cost). Loss of productivity was measured using human capital approach (calculated as; daily income/number of days of patients were unable to work). Medication cost was calculated based on the unit price of drug year 2015 and this information was obtained from the hospital’s administrative. Cost of phototherapy, lab tests and radiological procedure were estimated using the Ministry of Health’s Fee Act 1951 (revised 1982) for Ministry of Health hospitals (Hospital Kuala Lumpur, Hospital Pulau Pinang, Hospital Sultanah Bahiyah, and Hospital Sultanah Aminah)^28^ and charges posed by Universiti Kebangsaan Malaysia Medical Centre. Details of medication, lab tests and radiology were explained in the previous study.^29^ All were added to provide total cost of medication. Patient costing form provided primary data for patient out-of-pocket expenditures, transportation and time taken off work, for a duration of 6 month after being recruited into the study. The cost effectiveness was measured by the cost per PASI-75 and/or BSA <5 and/or DLQI ≤5 achieved. This was calculated by dividing the total cost by the number of patients who achieved this response. Then, a sensitivity analysis has been conducted to resolve any uncertainties behind the input parameters, by integrating variability in the results and producing confidence intervals for each strategy. This has been done to determine and evaluate the robustness of the outcomes towards variations in the final decision model. A scenario analysis based on 3 cases (ie, best, base, and worst case) has also been constructed by applying a 15% variation into both critical variable, cost and effectiveness (into average cost and effectiveness) and this was similar to the previous study done by Vañó-Galván et al.^11^ One-way analysis has also been conducted by applying ± 15% on every variable in the study such as cost of medication (ie, systemic, biologic), cost of lab tests and radiology, loss of productivity, probability of effectiveness (PASI-75 and/or BSA <5 and/or DLQI ≤5), cost of transportation and out-of-pocket expenses to determine the most sensitive parameter the model. Statistical analysis was performed using version 21.0 of the Statistical Package for Social Sciences (SPSS Inc.) and Microsoft Excel 2010 for the cost analysis.

## Results

**Figure 1:**
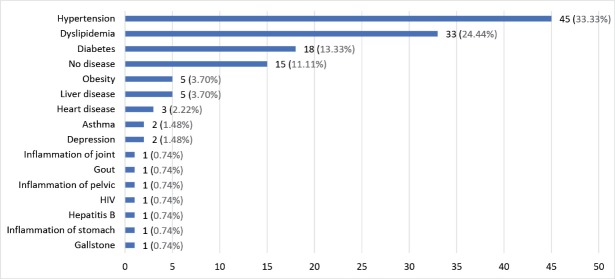
Comorbidity Among Patients.

A total of 90 moderate to severe psoriasis patients were included in the study. The demographic characteristics of the respondents are shown in Table 1. Majority of the respondents were male, 60 (66.7%), mean age was 45 ± 15 years old (range 19-84 years), 57 (53.3%), Malays, 56 (62.2%), have low educational background, 54 (60%), married, 60 (67.7%), employed, 49 (54.4%), having monthly income more than RM3000, 61 (67.8% and have mean ± standard deviation (SD) baseline PASI, BSA, and DLQI 16.02 ± 10.54, BSA 26.66 ± 21.72 and 13.87 ± 5.72 respectively. With respect to comorbidity, majority of them have hypertension, 45 (33.3%) (Figure 1).

**Table 1:** Demographic Profile of the Respondents (n = 90)

**Characteristics**	**No. (%)**
Gender	
Male	60 (66.7)
Female	30 (33.3)
Mean age ± SD	45 ± 15 (range 19-84)
Ethnicity	
Malays	56 (62.2)
Non-Malays	34 (8.8)
Education	
Low (no schooling, primary school, secondary school)	54 (60.0)
High (college/university)	36 (40.0)
Marital status	
Single	30 (33.3)
Married	60 (66.7)
Occupation	
Unemployed	35 (38.9)
Self-employed	6 (6.7)
Employed	49 (54.4)
Income	
RM 0-3000	29 (32.2)
RM >3000	61 (67.8)
Mean PASI baseline score	16.02±10.54
Mean BSA baseline score	26.66±21.72
Mean DLQI baseline score	13.87±5.72

Abbreviations: SD, standard deviation; RM, Malaysian Ringgit; PASI, psoriasis area severity index; BSA, body surface area; DLQI, dermatology life quality index.

**Figure 2:**
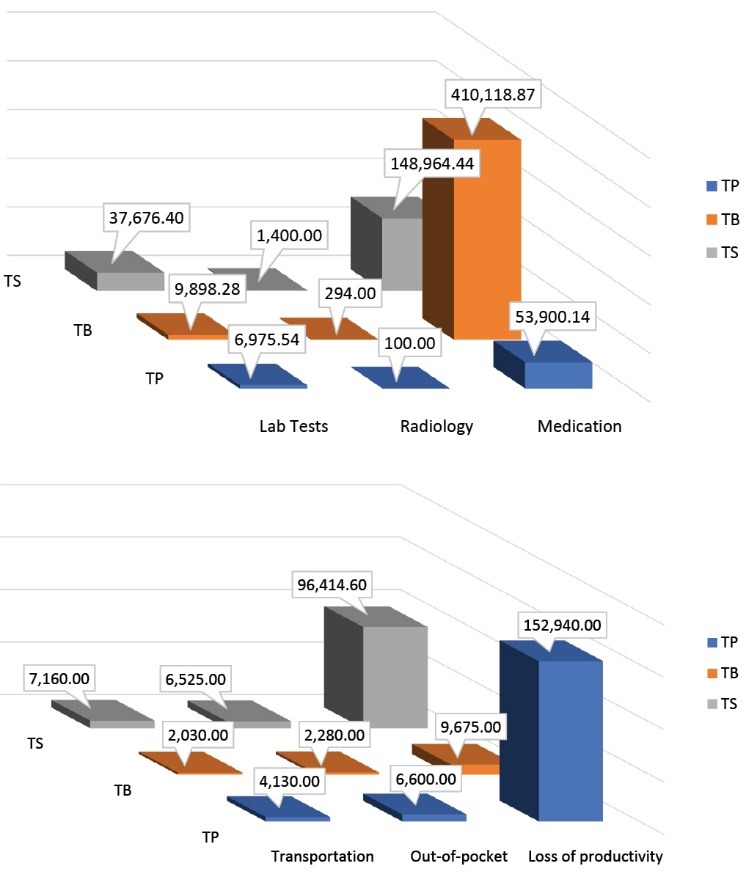
Costs From Patient’s Perspective According to Treatment. Abbreviations: TP, topical and phototherapy; TS, topical and systemic; TB, topical and biologic.

Table 2 presents the total costs associated with psoriasis treatments over a 6-month period. TB has been found to produce the highest total cost, value at RM434 296.15 (RM36 191.35/patient), followed by TS, valued at RM298 140.44 (RM4969.01/patient), and TP, valued at RM224 645.68 (RM12 480.32/patient) (Table 2). Figure 2 demonstrates cost according to the components for each modality. TB treatment has produced highest total cost of medication which was RM410 118.87 (RM34 176.57/patient), TS has generated highest total cost of lab tests and transportation which was RM37 676.40 (RM627.94/patient) and RM7160.00 (RM119.33), respectively. Meanwhile, TP treatment has yielded greatest loss of productivity cost which was RM152 940.00 (RM8496.67/patient). The result also indicates that there was statistically significant difference in cost of medication, lab tests, transportation and loss of productivity among 3 types of treatment options (Table 3).

**Table 2:** Overall Costs Associated With the Management of Moderate to Severe Psoriasis

**Treatment**	**Total cost (RM)**	**Cost/Patient (RM)**	**Percent**	***P *** **Value**
TP (n = 18)	224 645.68	12 480.32	23.5	.001
TS (n = 60)	298 140.44	4969.01	31.2	.001
TB (n = 12)	434 296.15	36 191.35	45.4	.001
Total (n = 90)	957 082.27	10 634.25		

Abbreviations: TP, topical and phototherapy; TS, topical and systemic; TB, topical and biologic; RM, Malaysian Ringgit.

**Table 3:** Mean Cost According to the Components

**Input**	**Treatment**			***P*** ** Value**
	**TP (n = 18)**	**TS (n = 60)**	**TB (n = 12)**	
	**Mean (SD)**	**Mean (SD)**	**Mean (SD)**	
Provider				
Medication^a^	2994.45 (1541.21)	2482.74 (2389.72)	34 176.57 (8809.28)	.00
Lab tests^a^	387.53 (312.90)	627.94 (291.30)	824.86 (466.85)	.00
Radiology	5.56 (RM16.70)	23.33 (41.65)	24.50 (22.92)	.164
Total mean cost (RM)	3387.54	3134.01	35 025.93	
Patient				
Out of pocket expenses	366.67 (810.41)	108.75 (254.65)	RM190.00 (401.50)	.09
Transportation^a^	229.44 (215.58)	119.33 (110.74)	169.17 (123.27)	.00
Loss of productivity^a^	8496.67 (7626.14)	1606.91 (4954.32)	806.25 (616.22)	.00
Total mean cost (RM)	9092.78	1834.99	1165.42	.00
Total costs (RM)	224 645.76	298 140.44	434 296.15	.001

Abbreviations: SD, standard deviation; RM, Malaysian Ringgit; TP, topical and phototherapy; TS, topical and systemic; TB, topical and biologic. ^a^ANOVA, significant at *P* < .05.

**Figure 3:**
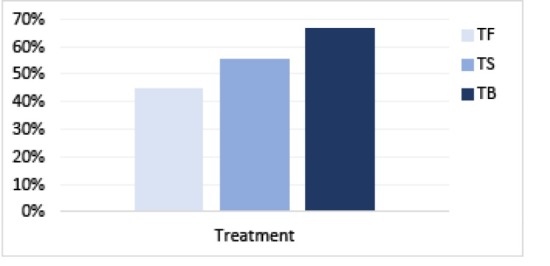
Effectiveness of the Treatment Modalities.
Note: Effectiveness = percentage or number of patients achieved PASI-75 and/or BSA <5 and/or DLQI ≤5. Abbreviations: TP, topical and phototherapy; TS, topical and systemic; TB, topical and biologic.

Next, Figure 3 illustrates the effectiveness of the treatments which was measured by the number of patients achieved PASI-75 and/or BSA <5 and/or DLQI ≤5. TB treatment has the highest number of patients achieved PASI-75 and/or BSA <5 and/or DLQI ≤5 which was 8 out of 12 patients (67.7%), followed by TS with 33 out of 60 patients (55.0%) and TP with 8 out of 18 patients (44.4%).

Table 4 shows the base-case results of the cost-effectiveness analysis from the societal perspective over a 6-month period. TS has been revealed as the most cost-effective modality as it yields the lowest cost per PASI-75 and/or BSA <5 and/or DLQI ≤5, valued at RM9034.56. This is followed by TP treatment, which is valued RM28 080.71, and finally, TB treatment, valued at RM54,287.02. The incremental cost effectiveness ratio (ICER) of TS compared to TP was RM2939.79 per additional patient with a PASI-75 and/or BSA <5 and/or DLQI ≤5 (calculated as follows; [RM298 140.44-RM224 645.68]/[33 patients with a PASI-75 and/or BSA <5 and/or DLQI ≤5, -8 patients with a PASI-75 and/or BSA <5 and/or DLQI ≤5]), whereas TB was dominated by TS with as denoted by negative ICER which was -RM5446.23 (calculated as follows: [RM434 296.15-RM298 140.44]/[8 patients with a PASI-75 and/or BSA <5 and/or DLQI ≤5–33 patients with a PASI-75 and/or BSA <5 and/or DLQI ≤5]).

**Table 4:** Base-Case Result of the Cost-Effectiveness

**Treatment**	**Total Cost (RM)**	**Effectiveness** ^a^ ** (%) (n)**	**Cost-Effectiveness** ^b^	**ICER**
TP	224 645.68	44.4 (8/18)	28 080.71	**-**
TS	298 140.44	55.0 (32/60)	9034.56	2939.79
TB	434 296.15	67.7 (8/12)	54 287.02	-5446.23

Abbreviations: TP, topical and phototherapy; TS, topical and systemic; TB, topical and biologic; RM, Malaysian Ringgit; ICER, incremental cost effectiveness ratio. ^a^Effectiveness (%) = percentage of patients achieved PASI-75 and/or BSA <5 and/or DLQI ≤5. ^b^Cost effectiveness = total cost divides by the number of patients achieved PASI-75 and/or BSA <5 and/or DLQI ≤5.

Then, a scenario analysis (as per Table 5) was undertaken with a 15% variation of cost and effectiveness, with the cost per PASI-75 and/or BSA <5 and/or DLQI ≤5 for TP regiment valued at RM9079.43 in the best-case scenario (mean cost reduced by a 15% and an effective response [50% (9/18)]), RM28 080.71 in the base case, (mean cost and effective response [44% (8/18)]) and RM36 906.08 in the worst case (mean cost increased by a 15% and effective response [39% (7/18)]). Meanwhile, the cost per PASI-75 and/or BSA <5 and/or DLQI ≤5 for TS in the best case was RM6668.93 (mean cost reduced by a 15% and effectiveness response [63% (38/60)]), RM9038.69 in the base case (mean cost and effective response [53% (32/60)]) and RM12 245.05 in worst case (mean cost increased by a 15% and effective response [47% (28/60)]). Kos per PASI-75 and/or BSA <5 and/or DLQI ≤5 for TB was RM41 016.86 in the best case (mean cost reduced by a 15% and effective response [75% (9/12)]), RM54 287.02 in the base case (average cost and effective response [67% (8/12)]) and RM71 348.65 in the worst care (mean cost increased by a 15% and effective response [58% (7/12)]).

**Table 5:** Scenario Analysis

**Treatment**	**Scenario (Cost-Effectiveness) (RM)**		
	**Best** ^a^	**Base** ^b^	**Worst** ^c^
TP	21 216.54	28 080.71	36 906.08
TS	6668.93	9034.56	12 245.05
TB	41 016.86	54 287.02	71 348.65

Abbreviations: TP, topical and phototherapy; TS, topical and systemic; TB, topical and biologic; RM, Malaysian Ringgit. ^a^Best case = average cost reduced to 15% and effectiveness response increased to 15%. ^b^Base case = average cost and effectiveness response. ^c^Worst case = average cost increased to 15% and effectiveness response reduced to 15%.

**Figure 4:**
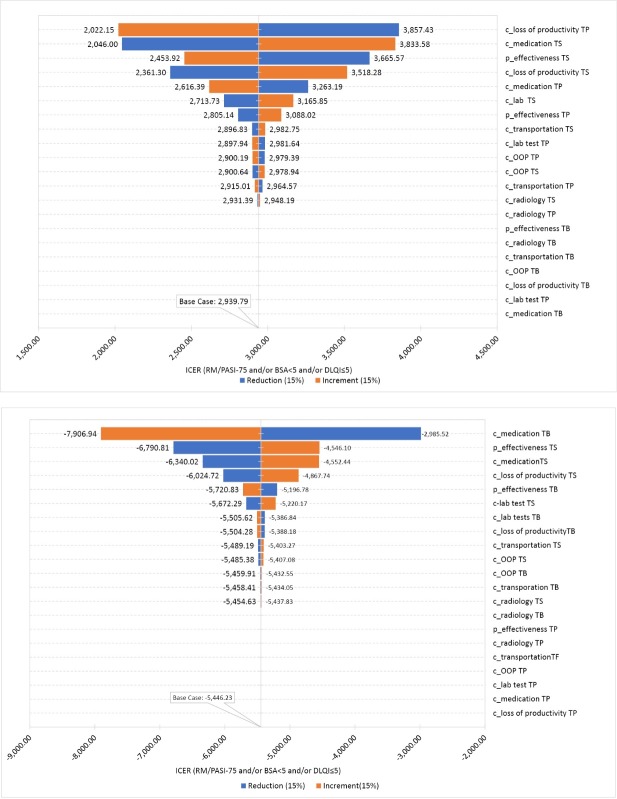
(A) Tornado Diagram Expressed in Terms of ICER Impact (TS vs. TP). (B) Tornado Diagram Expressed in Terms of ICER Impact (TB vs. TS).
Abbreviations: TS, topical and systemic; TP, topical and phototherapy; TB, topical and biologic; RM, Malaysian Ringgit; ICER, incremental cost effectiveness ratio; BSA, body surface area; PASI, psoriasis area severity index; DLQI, dermatology life quality index.

Figure 4A-B  illustrates a Tornado diagram expressed in ICER. Dotted lines correspond to the ICER value in the base-case scenario. One-way sensitivity analysis pointed out the cost of biologics and loss of productivity cost as the most sensitive parameter of the model.

## Discussion

This study has emerged as the first and pioneering economic evaluation in assessing the cost-effectiveness of 3 psoriasis treatments for moderate to severe psoriasis in Malaysia. Previous analyses of the cost-effectiveness of these interventions has yielded mixed results. Some evidence has suggested the biologic modality as the most cost-efficient option compared to other modalities.^30-32^ Meanwhile, other works have indicated that the systemic treatment has generated the lowest cost per PASI response^33^ whereas several studies have demonstrated phototherapy to be the most cost-effective regiment. Nevertheless, this particular work has suggested the TS treatment to be the most cost-effective modality, despite TB showing the best results in terms of effectiveness. This especially relevant as the cost-effectiveness analysis taking into account both cost and effectiveness in determining the most cost-effective strategy. The costs analysis in this study has demonstrated that the total cost of TS to be twice lesser than TB, whereas effectiveness difference has only been found to be at 13%. Hence, this indicates that the TS treatment modality is the most cost-effective regimen.

The inconsistent findings obtained in determining the most cost-effective treatment for moderate to severe psoriasis can be attributed to several factors. One of them includes different effectiveness measure, which is believed to be the main cause for the large variation in cost-effectiveness values displayed in this study versus Sizto et al.^35^ The latter work has found that systemic medication (eg, cyclosporine) has exhibited the lowest cost/quality-adjusted life year from the societal perspective in the United Kingdom, valued at RM165 441.90 (£25 135). This is in comparison with the biologic regiment, which is valued between RM245 408.27 to RM279 688 (£37 284-£42 492). In this particular study, the cost/PASI-75 and/or BSA <5 and/or DLQI ≤5 for systemic treatment has also been found to be less than the biologic treatment, valued at RM9038.69 compared to RM52 659.51 respectively. Furthermore, the duration of time taken for the study to be undertaken is also capable of influencing the results. Pearce et al^36^ have demonstrated the systemic agent to be the most cost-effective strategy in the United States, with the lowest cost/PASI-75 of RM2837.62 (US$623). However, that value is particularly less compared to this study that has yielded a value of RM9038.69 (US$2583.73). The previous study has calculated cost and effectiveness for a systemic agent during a 12-week time period, as opposed to this study that has measured four systemic medications (ie, methotrexate, cyclosporine, acitretin and sulphasalazine) for 24 weeks. Therefore, a longer duration is capable of affecting the outcome, as both overall costs and the probability of treatment success are both increased. This is also justified by the work by Cabello Zurita et al^37^ whereby approximately 33.3% of the patients being treated with methotrexate have achieved 75% reduction of psoriasis symptoms by week 12. The percentage has shown considerable increments to 34.9%, 44.7%, and 52.8% at week 16, 24, and 48 respectively.

The duration of the cost effectiveness study of the psoriasis treatment modalities defined in different works is also variable. Knight et al^38^ have assessed cost-effectiveness of psoriasis treatments for 10 years, Verma et al^39^ at 5 years, Villacorta et al^40^ at 3 years, Küster et al^30^ at 2 years, Ahn et al,^41^ and Salazar et al^42^ at 1 year. The time of horizon of less than 1 year was sustained by available evidence. Although a long-time horizon is preferred, data associated with to the long-term experience with several psoriasis therapies is still lacking. Such information includes the annual drop-out rates from treatment, the ‘remission’ period, the efficacy of subsequent lines of treatment, the cost and incidence of side effects and the risk of hospitalization.^43^

Furthermore, numerous studies focused on the economic evaluation of psoriasis treatments have typically compared 2 interventions only.^11,32,44-46^ Out of the 19 studies that have differentiated the cost-effectiveness of disparate treatment options, only few studies have opted to evaluate in terms of 3 interventions, namely systemic, biologic and phototherapy.^32-36^ Most of the recent analyses are generally dominated by studies evaluating the cost-effectiveness of biologic drugs.^40,43,47-51^ This evaluation is especially relevant as the biologic regiment is commonly known as the best intervention in treating moderate to severe psoriasis, offering high safety profile, fewer side effects and increased patient’s quality of life. Hence, a cost-effectiveness study is paramount to justify the need for biologic agents in the respective countries.

In this study, PASI-75 and/or BSA <5 and/or DLQI ≤5 has been considered as an outcome. These responses are widely used in studies involving psoriasis, with PASI, in particular, being underlined as the gold standard and meeting the criteria of methodological validity.^26,27,52,53^ Furthermore, the score has also been proven to be strongly correlated with BSA and DLQI.^56,57^ Previous studies have opted for utility measures, whereby the clinical outcome is converted to utility score using the EuroQol 5-dimensional questionnaire. Then, it is used to estimate the quality-adjusted life year. However, various evidence has demonstrated that PASI and DLQI responses to range between weak to moderately correlated with EuroQol 5-dimensional questionnaire.^58,59^ Hence, using utility values by means of PASI response has been linked to a high level of bias.^54^ Additionally, another important information elicited from the findings of this study is that the cost of medication (ie, biologic and systemic) is the most sensitive parameters. Similarly, various previous works have highlighted the biologic medications as the highest contributor towards the overall cost of medication,^54^ resulting in several-fold escalation of overall cost of treatments.^19,54,60^ Similarly, analytical trends in systemic psoriasis treatment costs have revealed that biologic medications to exceed general inflation, with an incremental rate for biologic agents of 120% for etanercept, 103% for adalimumab and 53% for ustekinumab during the period 2004-2014. In contrast, their average annual increment within the same period is 8.2% for etanercept, followed by 9.2% (adalimumab) and 11.0% (ustekinumab).^61^

Regardless, this study is also associated with several limitations. Firstly, the respondents have been recruited from 5 tertiary, government-run hospitals only and excluded patients who sought treatment in private clinics and hospitals. Hence, the data obtained may not be completely representative of all cases of moderate to severe psoriasis in Malaysia. Nevertheless, it has provided meaningful insight to clinicians anyway regarding resource utilization in managing psoriasis. Secondly, time duration utilised in this study is less than a year despite psoriasis being a long-term and chronic disease. Therefore, is important to establish a cost-effectiveness model that is capable of predicting changes and interruptions during treatment, as well as its effectiveness in many coming years. But, conducting and maintaining a long-term study is a very difficult task due to the high dropout rate and mid-treatment changes occurring. It is justifiable that high drop out rate could lead to selection bias that affects conclusion of the finding. Therefore, the findings of the study could be limited to the fact that TS is the most cost-effective treatment in Malaysia if majority of the patients are moderate psoriasis (PASI >10-20, BSA >10-30 and DLQI >10-20 as refers to the classification of disease severity by the guideline of the Management of Psoriasis Vulgaris in Malaysia).^6^ Thirdly, the associated side effect costs have also been excluded. Generally, the side effects of a treatment are very complex, especially for diseases involving many comorbidities like psoriasis. This renders costing calculations to be a demanding and challenging task, despite the inevitable importance of the role that side effects play during treatment decision-making.

## Conclusion

Treatment for moderate to severe psoriasis causes considerable direct and indirect costs. TB treatment exhibited highest effectiveness but, TS treatment is considered the most cost-effective strategy in Malaysia situation in where majority of the patients are moderate psoriasis. The important finding of this study is to guide policy makers to determine the first line treatment considering its cost and effectiveness for moderate to severe psoriasis in Malaysia, allows hospitals to justify and provide the essential capitals for further research and development as well as adoption of better treatment options. Future cost-effective analysis should provide information on the long-term experience with psoriasis interventions and manage the uncertainty associated with key drivers of the cost effectiveness of psoriasis treatments.

## Acknowledgements

The authors would like to express appreciation to the Ministry of Health and Head of Dermatology Department Universiti Kebangsaan Malaysia Medical Centre, Hospital Kuala Lumpur, Hospital Sultanah Aminah, Johor Bahru, Hospital Pulau Pinang and Hospital Sultanah Bahiyah, Alor Setar for granting this study as well as patients who participated in this study.

## Ethical issues

Medical Research and Ethics Committee (MREC) (NMRR:15-62925195).

## Competing interests

Authors declare that they have no competing interests.

## Authors’ contributions

NAA: conception and design, acquisition of data, analysis and interpretation of data, drafting the manuscript and statistical analysis. SS: conception and design, critical revision of the manuscript for important intellectual content, obtaining fund, supervision and administrative and material support. AI: conception and design, critical revision of the manuscript for important intellectual content, obtaining fund, supervision and administrative and material support. NMN: conception and design, critical revision of the manuscript for important intellectual content and administrative and material support.

## Authors’ affiliations

^1^Department of Community Health, Faculty of Medicine, Universiti Kebangsaan Malaysia, Selangor, Malaysia. ^2^Faculty of Business and Management, Universiti Teknologi MARA Selangor, Selangor, Malaysia. ^3^Department of Community Health, Universiti Kebangsaan Malaysia Medical Centre, Kuala Lumpur, Malaysia. ^4^Medical Department, Universiti Kebangsaan Malaysia Medical Centre, Kuala Lumpur, Malaysia.

## Key messages

**Implications for policy makers**

- Determination of the most cost-effective strategy for the moderate to severe psoriasis patients in Malaysia.
- Topical and biologic (TB) intervention exhibited highest total cost which was RM434 296.15 (US$124 144.50).
- Topical and systemic (TS) treatment was the most cost effectiveness treatment with the lowest cost per psoriasis area severity index (PASI)-75 and/or body surface area (BSA) <5 and/or dermatology life quality index (DLQI) ≤5 which was RM9034.56 (US$2582.55).
- TS appeared to the most cost-effective treatment in the situation where majority of the patients were moderate psoriasis (PASI >10-20, BSA >10-30 and DQLI >10-20).
- The findings will help policy makers in allocation of the resources for the betterment of the psoriasis management in Malaysia.

**Implications for public**

This is the first study conducted in Malaysia to measure cost, effectiveness and cost effectiveness of 3 psoriasis interventions namely; topical and phototherapy (TP), topical and systemic (TS), topical and biologic (TB). TP modality was associated with highest loss of productivity cost, RM152 940.00 (US$43 718.23) or 59% of the total productivity costs. TS incurred highest monitoring costs, which was RM37 676.40 (US$10 769.88) or 69% of the total lab tests cost. Meanwhile, TB yielded greatest cost of medication, RM410 118.87 (US$117 233.38) or 67% of the total medication cost. In terms of effectiveness, TB showed the highest (66.7%) while TS appeared to be the most cost-effective treatment with RM9,034.56 (US$2582.55)/psoriasis area severity index (PASI)-75 and/or body surface area (BSA) <5 and/or dermatology life quality index (DLQI) ≤5. The findings of this study will help policy-makers in determining appropriate resource allocation for psoriasis management in Malaysia as well as adoption of better strategy considering cost and effectiveness.

## References

[1] Rachakonda TD, Schupp CW, Armstrong AW (2014). Psoriasis prevalence among adults in the United States. J Am Acad Dermatol.

[2] Springate DA, Parisi R, Kontopantelis E, Reeves D, Griffiths CEM, Ashcroft DM (2017). Incidence, prevalence and mortality of patients with psoriasis: a UK population-based cohort study. Br J Dermatol.

[3] Ding X, Wang T, Shen Y (2012). Prevalence of psoriasis in China: a population-based study in six cities. Eur J Dermatol.

[4] Mohd Affandi A, Ngah Saaya N, Johar A, Muneer A, Hamid A. Annual Report of the Malaysian Psoriasis Registry 2007-2015. Vol 13. Kuala Lumpur; 2017.

[5] Thorleifsdottir R, Sigurdardottir S, Sigurgeirsson B (2017). Patient-reported outcomes and clinical response in patients with moderate-to-severe plaque psoriasis treated with tonsillectomy: a randomized controlled trial. Acta Derm Venereol.

[6] Ministry of Health. Management of Psoriasis Vulgaris. Kuala Lumpur; 2013.

[7] Carrascosa JM, Bonanad C, Dauden E, Botella R, Olveira-Martín A (2017). Psoriasis and nonalcoholic fatty liver disease on behalf of the systemic inflammation in psoriasis working group. Actas Dermosifiliogr.

[8] Gisondi P, Cazzaniga S, Chimenti S (2013). Metabolic abnormalities associated with initiation of systemic treatment for psoriasis: evidence from the Italian Psocare Registry. J Eur Acad Dermatol Venereol.

[9] Shah K, Mellars L, Changolkar A, Feldman SR (2017). Real-world burden of comorbidities in US patients with psoriasis. J Am Acad Dermatol.

[10] Singh S, Young P, Armstrong AW (2017). An update on psoriasis and metabolic syndrome: A meta-analysis of observational studies. PLoS One.

[11] Vañó-Galván S, Gárate MT, Fleta-Asín B (2012). Analysis of the cost effectiveness of home-based phototherapy with narrow-band UV-B radiation compared with biological drugs for the treatment of moderate to severe psoriasis. Actas Dermosifiliogr.

[12] Menter A, Korman NJ, Elmets CA (2009). Guidelines of care for the management of psoriasis and psoriatic arthritis. J Am Acad Dermatol.

[13] Alfageme Roldán F, Bermejo Hernando A, Calvo González JL, Marqués Sánchez P (2016). Cost Effectiveness of Treatments of Psoriasis with a PASI 75 and one Period of 12 Weeks. Rev Esp Salud Publica.

[14] Czarnecka-Operacz M, Sadowska-Przytocka A (2014). The possibilities and principles of methotrexate treatment of psoriasis - the updated knowledge. Postepy Dermatol Alergol.

[15] Oussedik E, Patel NU, Cash DR, Gupta AS, Feldman SR (2017). Severe and acute complications of biologics in psoriasis. G Ital Dermatol Venereol.

[16] Mansouri Y, Goldenberg G (2015). Biologic safety in psoriasis: Review of long-term safety data. J Clin Aesthet Dermatol.

[17] Puig L, Julià A, Marsal S (2014). The Pathogenesis and Genetics of Psoriasis. Actas Dermo-Sifiliográficas (English Ed).

[18] Burgos-Pol R, Martínez-Sesmero JM, Ventura-Cerdá JM, Elías I, Caloto MT, Casado MÁ (2016). Coste de la psoriasis y artritis psoriásica en cinco países de Europa: una revisión sistemática. Actas Dermosifiliogr.

[19] Spandonaro F, Ayala F, Berardesca E (2014). The cost effectiveness of biologic therapy for the treatment of chronic plaque psoriasis in real practice settings in Italy. BioDrugs.

[20] Korman NJ, Zhao Y, Pike J, Roberts J, Sullivan E (2015). Increased severity of itching, pain, and scaling in psoriasis patients is associated with increased disease severity, reduced quality of life, and reduced work productivity. Dermatol Online J.

[21] Schmitt J, Küster D (2015). Correlation between Dermatology Life Quality Index (DLQI) scores and Work Limitations Questionnaire (WLQ) allows the calculation of percent work productivity loss in patients with psoriasis. Arch Dermatol Res.

[22] Chan B, Hales B, Shear N (2009). Work-related lost productivity and its economic impact on Canadian patients with moderate to severe psoriasis. J Cutan Med Surg.

[23] Lewis-Beck C, Abouzaid S, Xie L, Baser O, Kim E (2013). Analysis of the relationship between psoriasis symptom severity and quality of life, work productivity, and activity impairment among patients with moderate-to-severe psoriasis using structural equation modeling. Patient Prefer Adherence.

[24] Li K, Armstrong AW (2012). A review of health outcomes in patients with psoriasis. Dermatol Clin.

[25] Zhang W, Islam N, Ma C, Anis AH (2015). Systematic review of cost-effectiveness analyses of treatments for psoriasis. Pharmacoeconomics.

[26] Baker C, Mack A, Cooper A (2013). Treatment goals for moderate to severe psoriasis: an Australian consensus. Australas J Dermatol.

[27] Mrowietz U, Kragballe K, Reich K (2011). Definition of treatment goals for moderate to severe psoriasis: a European consensus. Arch Dermatol Res.

[28] Ministry Of Health. Surat Pekeliling Bil. 1 Tahun 2014 - Garis Panduan Pelaksanaan Perintah Fi (Perubatan) (Kos Perkhidmatan). http://www.moh.gov.my/index.php/database_stores/store_view_page/31/257. Published 2014. Accessed January 13, 2019.

[29] Zhang W, Islam N, Ma C, Anis AH (2015). Systematic review of
cost-effectiveness analyses of treatments for psoriasis. Pharmacoeconomics.

[30] Küster D, Nast A, Gerdes S (2016). Cost-effectiveness of systemic treatments for moderate-to-severe psoriasis in the German health care setting. Arch Dermatol Res.

[31] D’Ausilio A, Aiello A, Daniel F, Graham C, Roccia A, Toumi M (2015). A cost effectiveness analysis of secukinumab 300 MG vs current therapies for the treatment of moderate to severe plaque psoriasis in Italy. Value Heal.

[32] Knight C, Mauskopf J, Ekelund M, Singh A, Yang S, Boggs R (2012). {C}ost-effectiveness of treatment with etanercept for psoriasis in Sweden. Eur J Heal Econ.

[33] D’Souza LS, Payette MJ, Song M, Lim HW (2015). Estimated cost efficacy of systemic treatments that are approved by the US Food and Drug Administration for the treatment of moderate to severe psoriasis. J Am Acad Dermatol.

[34] Staidle JP, Dabade TS, Feldman SR (2011). A pharmacoeconomic analysis of severe psoriasis therapy: a review of treatment choices and cost efficiency. Expert Opin Pharmacother.

[35] Sizto S, Bansback N, Feldman SRR, Willian MKK, Anis AHH (2009). Economic evaluation of systemic therapies for moderate to severe psoriasis. Br J Dermatol.

[36] Pearce DJ, Nelson AA, Fleischer AB, Balkrishnan R, Feldman SR (2006). The cost-effectiveness and cost of treatment failures associated with systemic psoriasis therapies. J Dermatolog Treat.

[37] Cabello Zurita C, Grau Pérez M, Hernández Fernández CP (2017). Effectiveness and safety of Methotrexate in psoriasis: an eight-year experience with 218 patients. J Dermatolog Treat.

[38] Knight C, Mauskopf J, Ekelund M, Singh A, Yang S, Boggs R (2012). Cost-effectiveness of treatment with etanercept for psoriasis in Sweden. Eur J Heal Econ.

[39] Verma S, Dharmarajan S, Yang Y (2010). PSS12 modeling the cost-effectiveness of ustekinumab for moderate to severe plaque psoriasis in US. Value Heal.

[40] Villacorta R, Hay JW, Messali A (2013). Cost effectiveness of moderate to severe psoriasis therapy with etanercept and ustekinumab in the United States. Pharmacoeconomics.

[41] Ahn CS, Gustafson CJ, Sandoval LF, Davis SA, Feldman SR (2013). Cost effectiveness of biologic therapies for plaque psoriasis. Am J Clin Dermatol.

[42] Salazar A, Aguirre A, Flores R (2016). Cost-effectiveness analysis of ustekinumab for the treatment of adults with mild-to-moderate plaque psoriasis refractory to other biologic agents in Mexico. Value Heal.

[43] Armstrong AW, Betts KA, Signorovitch JE (2018). Number needed to treat and costs per responder among biologic treatments for moderate-to-severe psoriasis: a network meta-analysis. Curr Med Res Opin.

[44] de Argila D, Rodríguez-Nevado I, Chaves a (2007). Cost-Effectiveness Analysis Comparing Methotrexate With PUVA Therapy for Moderate—Severe Psoriasis in the Sanitary Area of Badajoz. Actas Dermo-Sifiliográficas (English Ed).

[45] Colombo G, Altomare G, Peris K (2008). Moderate and severe plaque psoriasis: cost-of-illness study in Italy. Ther Clin Risk Manag.

[46] Heinen-Kammerer T, Daniel D, Stratmann L, Rychlik R, Boehncke W-H (2007). Cost-effectiveness of psoriasis therapy with etanercept in Germany. J Dtsch Dermatol Ges.

[47] Johansson EC, Hartz S, Kiri SH, Kumar G, Svedbom A (June 2018). Cost-effectiveness analysis of sequential biologic therapy with ixekizumab versus secukinumab as first-line treatment of moderate-to-severe psoriasis in the UK. J Med Econ.

[48] Augustin M, McBride D, Gilloteau I, O’Neill C, Neidhardt K, Graham CN (2018). Cost-effectiveness of secukinumab as first biologic treatment, compared with other biologics, for moderate to severe psoriasis in Germany. J Eur Acad Dermatology Venereol.

[49] Klijn SL, van den Reek JMPA, van de Wetering G, van der Kolk A, de Jong EMGJ, Kievit W (2018). Biologic treatment sequences for plaque psoriasis: a cost-utility analysis based on 10 years of Dutch real-world evidence from BioCAPTURE. Br J Dermatol.

[50] Igarashi A, Kuwabara H, Fahrbach K, Schenkel B (2013). Cost-efficacy comparison of biological therapies for patients with moderate to severe psoriasis in Japan. J Dermatolog Treat.

[51] Pan F, Brazier NC, Shear NH, Jivraj F, Schenkel B, Brown R (2011). Cost utility analysis based on a head-to-head Phase 3 trial comparing ustekinumab and etanercept in patients with moderate-to-severe plaque psoriasis: a Canadian perspective. Value Health.

[52] Bronsard V, Paul C, Prey S (2010). What are the best outcome measures for assessing quality of life in plaque type psoriasis? A systematic review of the literature. J Eur Acad Dermatology Venereol.

[53] Hägg D, Sundström A, Eriksson M, Schmitt-Egenolf M (2017). Severity of psoriasis differs between men and women: a study of the clinical outcome measure psoriasis area and severity index (PASI) in 5438 Swedish register patients. Am J Clin Dermatol.

[54] Riveros BS, Ziegelmann PK, Correr CJ (2014). Cost-effectiveness of biologic agents in the treatment of moderate-to-severe psoriasis: a Brazilian public health service perspective. Value Heal Reg Issues.

[55] Cohen S, Baron S, Archer C, British Association of Dermatologists and Royal College of General Practitioners (2012). Guidance on the diagnosis and clinical management of psoriasis. Clin Exp Dermatol.

[56] Walsh JA, McFadden M, Woodcock J (2013). Product of the Physician Global Assessment and body surface area: a simple static measure of psoriasis severity in a longitudinal cohort. J Am Acad Dermatol.

[57] Mattei PLL, Corey KCC, Kimball ABB (2014). Psoriasis Area Severity Index (PASI) and the Dermatology Life Quality Index (DLQI): the correlation between disease severity and psychological burden in patients treated with biological therapies. J Eur Acad Dermatol Venereol.

[58] Blome C, Beikert FC, Rustenbach SJ, Augustin M (2013). Mapping DLQI on EQ-5D in psoriasis: transformation of skin-specific health-related quality of life into utilities. Arch Dermatol Res.

[59] Herédi E, Rencz F, Balogh O (2014). Exploring the relationship between EQ-5D, DLQI and PASI, and mapping EQ-5D utilities: a cross-sectional study in psoriasis from Hungary. Eur J Heal Econ.

[60] Driessen RJB, Bisschops LA, Adang EMM, Evers AW, Van De Kerkhof PCM, De Jong EMGJ (2010). The economic impact of high-need psoriasis in daily clinical practice before and after the introduction of biologics. Br J Dermatol.

[61] Beyer V, Wolverton SE (2010). Recent trends in systemic psoriasis treatment costs. Arch Dermatol.

